# Integration of multiple networks and pathways identifies cancer driver genes in pan-cancer analysis

**DOI:** 10.1186/s12864-017-4423-x

**Published:** 2018-01-06

**Authors:** Claudia Cava, Gloria Bertoli, Antonio Colaprico, Catharina Olsen, Gianluca Bontempi, Isabella Castiglioni

**Affiliations:** 1 Institute of Molecular Bioimaging and Physiology, National Research Council (IBFM-CNR), Via F.Cervi 93, 20090 Milan, Segrate-Milan Italy; 2 Interuniversity Institute of Bioinformatics in Brussels (IB)2, 1050 Brussels, Belgium; 3 Machine Learning Group (MLG), Department d’Informatique, Universite libre de Bruxelles (ULB), 1050 Brussels, Belgium

**Keywords:** Genes, Pathways, Multi-networks, Pan-cancer

## Abstract

**Background:**

Modern high-throughput genomic technologies represent a comprehensive hallmark of molecular changes in pan-cancer studies. Although different cancer gene signatures have been revealed, the mechanism of tumourigenesis has yet to be completely understood. Pathways and networks are important tools to explain the role of genes in functional genomic studies. However, few methods consider the functional non-equal roles of genes in pathways and the complex gene-gene interactions in a network.

**Results:**

We present a novel method in pan-cancer analysis that identifies de-regulated genes with a functional role by integrating pathway and network data.

A pan-cancer analysis of 7158 tumour/normal samples from 16 cancer types identified 895 genes with a central role in pathways and de-regulated in cancer.

Comparing our approach with 15 current tools that identify cancer driver genes, we found that 35.6% of the 895 genes identified by our method have been found as cancer driver genes with at least 2/15 tools.

Finally, we applied a machine learning algorithm on 16 independent GEO cancer datasets to validate the diagnostic role of cancer driver genes for each cancer. We obtained a list of the top-ten cancer driver genes for each cancer considered in this study.

**Conclusions:**

Our analysis 1) confirmed that there are several known cancer driver genes in common among different types of cancer, 2) highlighted that cancer driver genes are able to regulate crucial pathways.

**Electronic supplementary material:**

The online version of this article (10.1186/s12864-017-4423-x) contains supplementary material, which is available to authorized users.

## Background

Although an increasing number of disease biomarkers have been identified through high-throughput data, their reproducibility and overlap are poor. This poor reproducibility is possibly due to the fact that individual biomarkers are often selected without considering their metabolic role in terms of their cellular function.

Many studies have thus hypothesized that a more reproducible method may be to analyze gene expression profiles over functional pathways that express different cellular functions (e.g. i.e. cell cycle, apoptosis, proliferation) [1, 2]. Databases such as Gene Ontology [3], Reactome [4], the Kyoto Encyclopedia of Genes and Genomes (KEGG) [5], and Biocarta [6] describe the different cellular functions (pathways) as exploited by a list of genes. However, this functional pathway representation attributes the same functional significance to each gene in the list without considering the impact of gene interactions in performing this function.

What kinds of connections are there among genes in functional pathways? Some tools, such as GeneMania [7], describe the biological relationships among the cellular components, i.e. physical interactions, genetic interactions, shared-protein functional domains, or the co-localization of molecules. These connections identify those gene regulatory networks that play crucial roles in many key biological processes, such as cell differentiation, metabolism, cell cycles, and signal transduction.

Establishing the role of a gene within pathways and networks facilitates a multi-layered description of its functional role in both physiological and pathological conditions, and enables the network drivers to be identified [8]. When a pathological process is ongoing, the dynamics of pathways and networks are altered. Thus, the integration of networks with pathways can help to describe these dynamics and to identify the key network drivers with a functional role in the onset and progression of a disease. A perturbation of the expression level of a key network driver should have a larger impact on a pathway function compared to a non-key network driver, and on the network itself because of the connections between this gene and its downstream effectors.

Only a few methods have defined indexes that measure how central the role of a gene is in a functional pathway in terms of biological networks [9, 10]. These indexes could help to identify genes with key biological relationships within a functional pathway [9, 10], and to identify key network drivers. For example, in a graph analysis of a biological network, the degree centrality [9, 10] quantifies the number of interactions (edges) connected to a gene (node).

Several studies have shown that the absence of mutations in genes with a high degree centrality is vital for organism survival [11]. Indeed, the degree centrality can identify genes with a key role in the functional pathway [7, 8]. Degree centrality has been used by Fang [9] in an approach called “Gene Association Network-based Pathway Analysis” (GANPA) in order to assign a weight for each gene within pathways. The assignment of weights for each gene in a pathway is based on to its relative association with genes inside and outside the pathway in a functional association network, based on protein-protein interactions, co-annotations, and co-expression. GANPA has also been proposed as a tool for the Functional Category Score (FCS), where a weighted gene is integrated with an expression change value to detect pathways with significant expression changes.

Dong et al. [10] adopted a similar approach to GANPA and developed a new tool for Over-Representation Analysis (ORA) called "functional Link Enrichment of Gene Ontology or gene sets" (LEGO). The main differences between ORA and FCS are: 1) FCS uses profiles of gene expression values, whereas ORA only considers the genes of interest, 2) the statistical test for FCS is based on the gene set, whereas ORA uses the interesting genes [10]. The aim of both GANPA and LEGO is to identify the relevant pathways of a particular condition.

The cancer research community refers to ‘cancer driver’ genes as genes whose perturbation (in the expression levels or in the sequence) confers a selective advantage to tumour growth [12]. Cancer driver genes need to be distinguished from ‘passenger’ genes, i.e. those genes whose mutation does not give any fitness advantage to the tumour [13]. In cancer, driver genes are those that accumulate mutations, or those that are differentially expressed in tumours vs normal samples (differentially expressed genes) [14, 15]. Both cases could lead to cancer initiation and development. Several studies have demonstrated that driver genes and driver mutations tend to accumulate in a limited number of cellular pathways, in which these driver genes have a central role [16–18].

Here, we propose an application of the GANPA/LEGO approach for the integrative analysis of multi-networks with multi-pathways with a novel purpose with respect to previous studies [9, 10]. Our aim was to reveal those network drivers that control key biological processes in a pan-cancer study.

In our approach, the GANPA/LEGO method was also extended by integrating biological multiplex networks. We thus defined key cancer network drivers as those cancer drivers that are simultaneously highly connected in at least two interaction types.

From an analysis of 7158 tumour/normal samples of 16 cancer types, we identified 895 differentially expressed genes with a central role in pathways and networks. These genes are deregulated in cancer with a reduced False Discovery Rate (FDR) compared to that obtained by commonly used differential expression analyses. For each cancer type, we also obtained a list of the top 10 cancer drivers able to classify normal versus tumour samples, with a high performance in independent datasets.

## Methods

### The computational approach

We used a modified version of the GANPA/LEGO algorithm [9, 10] to compute: 1) the degree centrality of genes inside networks (*d*_*N*_), and 2) the degree centrality of genes inside pathways (*d*_*P*_), as follows.

In the first step, given the gene *i* within the network N with m genes, we calculated degree centrality *di*_*N*_ as the number of neighbor genes belonging to N to which the gene *i* is directly linked.

In the second step, given gene *i* within pathway P with k genes, we then calculated degree centrality *di*_*P*_ considering only network interactions among gene *i* and the other genes in the networks belonging to pathway P. In this step by integrating the information of the network *N* within pathway P, we obtained a selection of interacting genes according to the network *N.*

Then, we computed degree centrality expected *di*_*E*_ by assuming equal probability for the existence of edges between nodes (*di*_*N*_*/m = di*_*E*_*/k*, [9, 10]). Thus,

$$ {di}_E={di}_N\ x\ k/m $$

We defined a gene as a ‘network driver’ in the pathway P, when, in at least two networks involving gene *i*, its *di*_*P*_, normalized to the size of the pathway (*k)*, is higher than *di*_*E*_, according to eq. 1:

1 $$ {di}_P/k>{di}_E $$

The hypothesis is that if one gene is functionally linked (according to the functional networks) with more genes in the pathway than expected, its role is functionally central in that pathway.

**Fig. 1:**
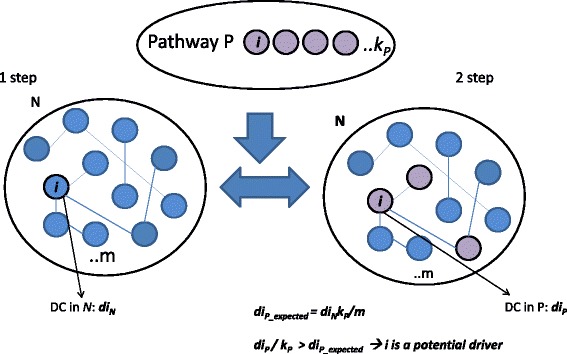
The computational approach. The first step involves a network *N* (e.g. physical interaction) of size *m* and for each gene, *i* in *N* the algorithm calculates its degree centrality, DC (*di*_*N*_). The second step involves a list of functional pathways (e.g. pathway *P)* and for each gene *i,* the DC *(di*_*p*_*)* is calculated using the information on interacting genes from *N.* For the assumption of equal probability for existing edges between nodes, the algorithm calculates the expected DC of gene *i* in the pathway *P*. If the DC observed for the gene *i (di*_*p*_*)* is higher than expected *(di*_*p_expected*_*)*, *i* could be a potential driver in the pathway *P*

Figure 1 shows the proposed procedure. The code is made available in the StarBioTrek package (http://bioconductor.org/packages/release/bioc/html/StarBioTrek.html).

### Pathways and networks

Using the KEGGREST [19] and StarBioTrek [20] packages, we downloaded 307 pathways from the KEGG database [5], which includes lists of genes grouped by functional role (e.g. cell cycle, apoptosis, proliferation). Different types of validated gene-gene or protein-protein networks, which include physical interaction, genetic interaction, shared protein domains, co-localization, and functional reaction interactions, were downloaded using SpidermiR [21].

### Differentially expressed genes

We applied our approach to cancer datasets obtained from the Cancer Genome Atlas (TCGA) [22]. We used the Illumina HiSeq RNAseqv2 of 7158 tumour/normal samples from 16 cancer types (see Table 1). We used the TCGAbiolinks package [23] and the TCGA Workflow [24] to download and process Level 3 TCGA gene expression data with platform RNAseqv2. The processing steps that were applied involve within-lane normalization procedures to adjust for GC-content effects on read counts and between-lane normalization procedures to adjust for distributional differences between lanes using the EDASeq package [25] as reported in [23, 24].

**Table 1:** Cancer types and number of samples for tumoural and normal tissues from TCGA database

Cancer Type	TCGA ID Data	No. Tumoural samples	No. Normal samples
Bladder Urothelial Carcinoma	BLCA	408	19
Breast invasive carcinoma cancer	BRCA	1097	114
Colon adenocarcinoma	COAD	286	41
Esophageal carcinoma	ESCA	184	11
Head and Neck squamous cell carcinoma	HNSC	520	44
Kidney Chromophobe	KICH	66	25
Kidney renal clear cell carcinoma	KIRC	533	72
Kidney renal papillary cell carcinoma	KIRP	290	32
Liver hepatocellular carcinoma	LIHC	371	50
Lung adenocarcinoma	LUAD	515	59
Lung squamous cell carcinoma	LUSC	503	51
Prostate adenocarcinoma	PRAD	497	52
Rectum adenocarcinoma	READ	94	10
Stomach adenocarcinoma	STAD	415	35
Thyroid carcinoma	THCA	505	59
Uterine Corpus Endometrial Carcinoma	UCEC	176	24

For each cancer type, we performed a differential expression analysis (DEA) between two classes, normal vs tumoural, using TCGAbiolinks [23, 24], identifying differentially expressed genes (DEGs) (logFC > 1, logFC < −1, FDR < 0.01) [23, 24].

### Application of our computational approach to the identification of cancer-specific drivers

We applied the computational approach described above to the study of key network drivers in cancer. For each cancer type, we computed *d*_*N*_, *d*_*P*_, and *di*_*E*_ of DEGs and selected cancer-specific network drivers accordance with (Eq. 1), which are DEGs with high degree centrality in networks with a functional role in the onset and progression of cancer.

In order to visualize the results obtained for each network and all possible combinations between different networks, we constructed a Venn diagram [26].

For each cancer type, we computed the number of cancer driver DEGs shared with all network drivers. We compared FDR, which measures the probability that a gene is a false positive DEG, between DEGs obtained by DEA and our cancer-specific network driver DEGs obtained in accordance with (Eq. 1) for each cancer type.

### Benchmarking validation

To verify our method, we compared our results and those obtained by other well-validated computational methods used to identify cancer driver genes, such as ActiveDriver [27], Dendrix [28], MDPFinder [29], Simon [30], NetBox [31], OncodriveFM [32], MutsigCV [33], MeMo [34], CoMDP [35], DawnRank [36], DriverNet [37], e-Driver [38], iPAC [39], MSEA [40], and OncodriveCLUST [41].

We applied DriverDB [42] to obtain results from all these algorithms and the cancer datasets considered by our computation approach.

### In silico validation

In silico validation analysis using our cancer drivers was performed using independent datasets. For each cancer, gene expression data were obtained from the GEO database (see Table 2). GEO datasets were analyzed using MoonlightR [43]. The processing steps included a normalization procedure (quantile analysis) and a log transformation using GEO2R as performed in [44].

**Table 2:** Independent datasets with number of tumoural and normal samples for each cancer type

Cancer Type	GEO ID Data	No. Tumoural samples	No. Normal samples
Bladder Urothelial Carcinoma	GSE13507	165	10
Breast invasive carcinoma cancer	GSE39004	61	47
Colon adenocarcinoma	GSE41657	25	12
Esophageal carcinoma	GSE20347	17	17
Head and Neck squamous cell carcinoma	GSE6631	22	22
Kidney Chromophobe	GSE15641	6	23
Kidney renal clear cell carcinoma	GSE15641	32	23
Kidney renal papillary cell carcinoma	GSE15641	11	23
Liver hepatocellular carcinoma	GSE45267	46	41
Lung adenocarcinoma	GSE10072	58	49
Lung squamous cell carcinoma	GSE33479	14	27
Prostate adenocarcinoma	GSE6919	81	90
Rectum adenocarcinoma	GSE20842	65	65
Stomach adenocarcinoma	GSE2685	10	10
Thyroid carcinoma	GSE33630	60	45
Uterine Corpus Endometrial Carcinoma	GSE10072	53	11

We developed a Random Forest (RF) classification model using R-package [45]. The model was used to classify the considered tumour versus normal samples using the gene expression levels of our cancer driver genes.

Receiver Operating Characteristic **(**ROC) curves and Area Under Curve (AUC) were estimated for each gene belonging to the cancer-specific driver DEGs by a cross-validation method (k-fold cross-validation, k = 10). We adopted the following parameters: mtry (number of variables randomly sampled as candidates at each split) = sqrt(p), p being the number of variables in the matrix of data; ntree (number of trees grown) = 500. We then created a list of the top ten cancer drivers with the best AUC performance for each cancer type.

## Results

### Network driver genes

**Fig. 2:**
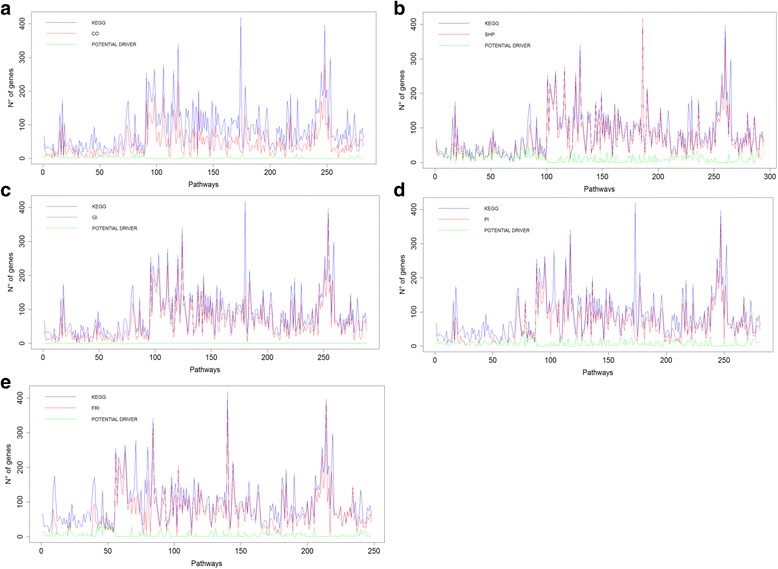
For each pathway (x axis), the following results are shown: the number of genes (y axis) in the original KEGG data (blue line), genes with a direct interaction (red line, **a** co-localization, CO; **b** shared protein domain, SHP; **c** genetic interaction, GI; **d** physical interaction, PI; and **e** functional reaction interactions, FRI) and the results of our computational method (green line, potential driver genes)

We applied our method for each functional network considered for 307 KEGG functional pathways (Fig. 2). The network that includes genes with genetic interaction found the lowest number of potential gene drivers, (50 genes). On the other hand, the network that includes proteins with shared protein domains found the highest number of potential driver genes, (1922 genes). Furthermore, our algorithm found 468 potential genes drivers for co-localization, 1402 for physical interaction, and 974 for functional reaction interactions.

Additional file 1 shows the potential driver genes for each pathway and network.

**Fig. 3:**
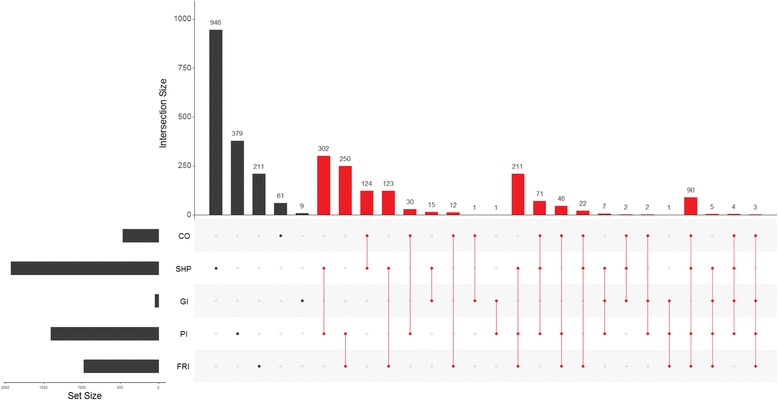
Potential driver genes obtained by our algorithm for each network. CO: co-localization, SHP: shared protein domain, GI: genetic interaction, PI: physical interaction, and FRI: functional reaction interactions. Driver genes found in at least two networks are shown in red

To focus on the network driver genes, we constructed a Venn diagram in which we plotted the list of potential gene drivers (y axis) and selected networks/intersecting networks (*x* axis) (Fig. 3). The results obtained by our method applied to the different networks highlighted 1322 common genes in at least two networks (Additional file 2).

As shown in Fig. 3 we obtained 102 network driver genes that were present in at least four networks, and there were no genes in any of the five networks considered.

### Cancer-specific driver DEGs

For each cancer type, with our approach we found the number of DEGs obtained by DEA from TCGA data (Table 3). For each cancer type we identified from all the DEGs the number of cancer-specific driver genes (cancer drivers with high degree centrality) (Table 3). We found 895 cancer-specific driver DEGs, in common among network drivers (1322) and DEGs (Table 3), i.e. 67% of all network driver genes. Additional file 3 shows the list of 895 genes.

**Table 3:** In each cancer type, the table shows the number of differentially expressed genes (DEGs) obtained by differential expression analysis from TCGA data, and for those genes the table shows the number of cancer driver DEGs and % of cancer driver DEGs with respect to 1322 driver genes

Cancer Type	TCGA ID Data	DEGs	Cancer driver DEGs	# cancer driver DEGs/# of driver genes (%)
Bladder Urothelial Carcinoma	BLCA	2937	217	16%
Breast invasive carcinoma cancer	BRCA	3390	249	19%
Colon adenocarcinoma	COAD	3788	289	22%
Esophageal carcinoma	ESCA	2525	229	17%
Head and Neck squamous cell carcinoma	HNSC	2973	225	17%
Kidney Chromophobe	KICH	4355	330	25%
Kidney renal clear cell carcinoma	KIRC	3618	307	23%
Kidney renal papillary cell carcinoma	KIRP	3748	294	22%
Liver hepatocellular carcinoma	LIHC	3043	238	18%
Lung adenocarcinoma	LUAD	3498	257	19%
Lung squamous cell carcinoma	LUSC	4984	399	30%
Prostate adenocarcinoma	PRAD	1860	113	8%
Rectum adenocarcinoma	READ	3628	273	20%
Stomach adenocarcinoma	STAD	2622	200	15%
Thyroid carcinoma	THCA	1994	130	9%
Uterine Corpus Endometrial Carcinoma	UCEC	4183	332	25%

**Fig. 4:**
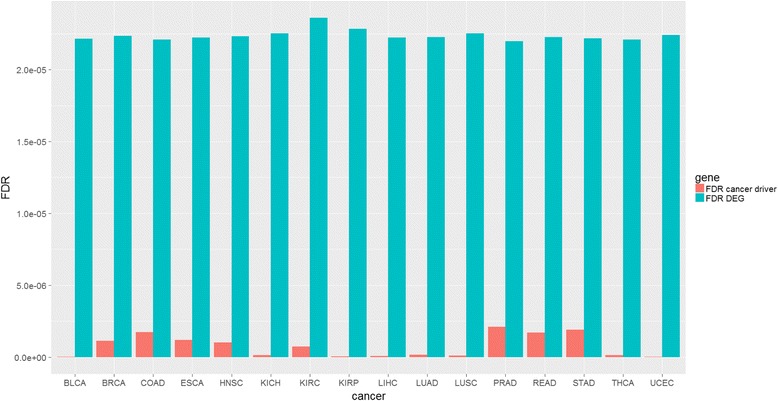
False Discovery Rate (FDR) of differentially expressed genes and cancer drivers obtained by our approach (red) and by gene expression analysis (light blue) for each cancer type

It should be noted that our computational approach found cancer driver DEGs with lower FDRs than that obtained by only DEA (Fig. 4).

**Fig. 5:**
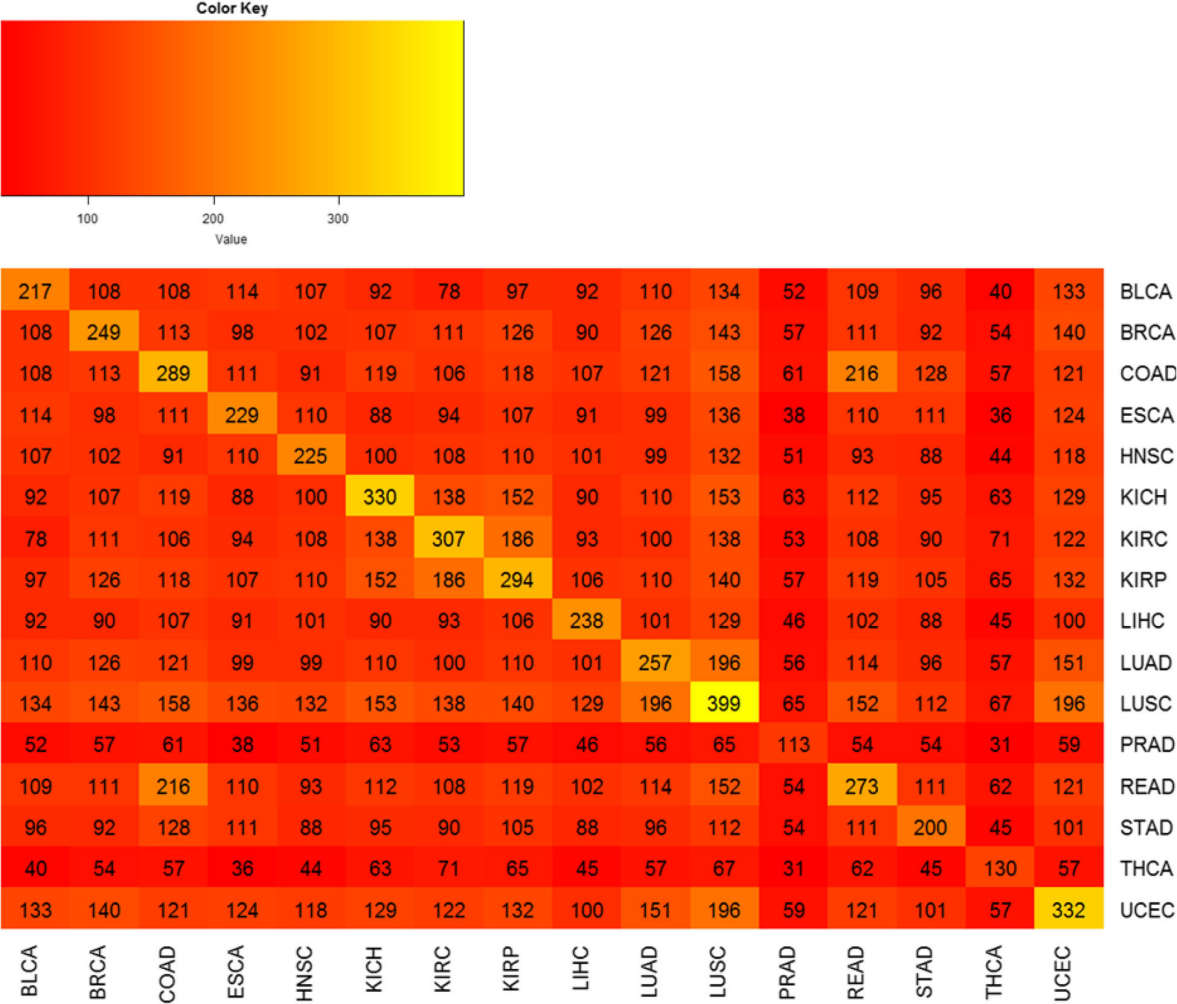
Cancer driver genes shared between two different cancer types

In order to highlight the number of cancer driver DEGs with high degree centrality in common between two different cancer types, we generated the heat map shown in Fig. 5.

Bladder urothelial carcinoma has 134 key cancer driver DEGs in common with lung squamous cell carcinoma, 196, 151 and 140 in common between lung squamous cell carcinoma and uterine corpus endometrial carcinoma, lung adenocarcinoma and uterine corpus endometrial carcinoma, and breast invasive carcinoma cancer and uterine corpus endometrial carcinoma, respectively.

The heat map in Fig. 5 shows that lung squamous cell carcinoma had the highest number of cancer-specific driver DEGs with high-degree centrality (399), while prostate adenocarcinoma had the lowest number (113).

### Benchmarking validation

A good overlap (319 genes out of 895, 35.6%) was found between our computational approach and the other well-validated computational methods considered in this study (Additional file 4). 10%, 6.8%, 3.2%, 1.9% and 1.1% was the percentage of overlap with three, four, five, six and seven methods, respectively (Table 4).

**Table 4:** Number of driver genes shared in common between our driver genes and the other tools. Genes in common with at least two, three, four, five, six and seven different methods are shown

Methods	# of common genes/# of our cancer driver DEGs	Percentage
2/15	319/895	35.6%
3/15	90/895	10%
4/15	61/895	6.8%
5/15	29/895	3.2%
6/15	17/895	1.9%
7/15	10/895	1.1%

Other tools provided a better overlap with two of the other well-validated computational methods. The percentage of overlapping ranged from about 20% (iPAC), 50% (CoMDP and MSEA), to 90% (MDPFinder and MeMo) with a mean of 76%. CoMDP, MSEA, MDPFinder and MeMo found in total 264 genes of the 319 obtained by our method. Thus, our method was able to find 55 genes out of 319 (17.2%), which had not been found using these four tools, and 32 genes out of 895 (3.6%) which had not been found using the other 15 tools (ALDH3B1, BIRC5, CFB, CLPS, COX7A2, COX7B, DNA2, GABARAPL1, GADD45B, GAS1,GNG2, GNG7, GNRH1, GSTT1, IDO1, LSM7, LTC4S, NAIP, PAFAH1B3, PIP5KL1, PNP, POLD4, PPP1R1B, PPP1R3E,PRIM2, PSME2, RFXANK, RPA3, RPP25, TUBA1A, VAMP2 and VAMP8). Although some of the other tools were able to find new cancer driver genes (MSEA found 83, CoMDP 6), the two tools that obtained the best overlap (MDPFinder and MeMo) were not able to discover new cancer driver genes.

### In silico validation

**Fig. 6:**
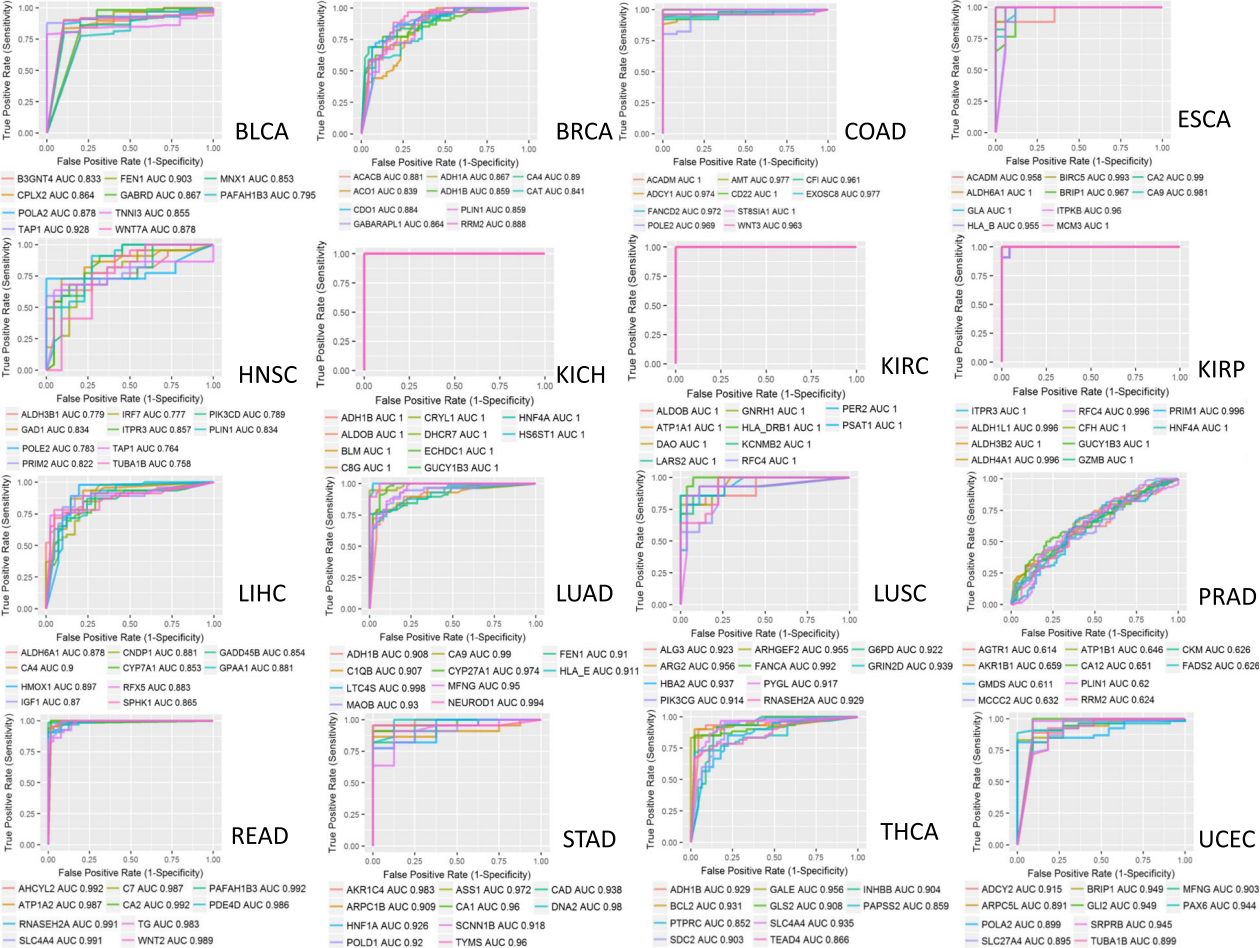
ROC Curves and AUC values for the top ten driver cancer-specific genes

Using independent GEO datasets, we calculated the performance of our method in the classification of cancer versus normal samples, for each cancer type (Fig. 6). ROC curves and AUC values are shown for the top ten cancer-specific driver DEGs (with high-degree centrality) with the highest AUC performance. The genes TAP1 and FEN1 achieved the best AUC performance in bladder urothelial carcinoma (AUC = 0.928 and AUC = 0.903), CA4 and RRM2 were the best predictors in breast invasive carcinoma cancer (AUC = 0.89 and AUC = 0.88), ACADM and CD22 in colon adenocarcinoma (AUC = 1), ALDH6A1, GLA and MCM3 in esophageal carcinoma (AUC = 1), GAD1 and PLIN1 in head and neck squamous cell carcinoma (AUC = 0.83, and AUC = 0.83).

The best performances were obtained in kidney chromophobe, kidney renal clear cell carcinoma and kidney renal papillary cell carcinoma datasets. Top ten genes for these cancers almost always obtained AUC = 1 (Fig. 6). In kidney chromophobe, the mean AUC for the genes, ranked between 11 and 50, was 0.96 and for those genes, ranked between 51 and 100, the AUC was 0.83. In kidney renal clear cell carcinoma mean AUC for the genes with a rank between 11 and 50 was 0.97, and for those ranked between 51 and 100, the AUC was 0.91. In kidney renal papillary cell carcinoma, the mean AUC for the genes ranked between the 11th and the 50th position was 0.96, and for those ranked between the fifty-one and the cent position was 0.87 (Table 5).

**Table 5:** Mean AUC for genes ranked between 11 and 50 and from 51 to 100 for kidney chromophobe, kidney renal clear cell carcinoma and kidney renal papillary cell carcinoma

	11–50	51–100
Kidney chromophobe	Mean AUC = 0.96	Mean AUC = 0.83
Kidney renal clear cell carcinoma	Mean AUC = 0.97	Mean AUC = 0.91
Kidney renal papillary cell carcinoma	Mean AUC = 0.96	Mean AUC = 0.87

CA4 and HMOX1 achieved the best AUC performance (AUC = 0.9 and AUC = 0.89) in liver hepatocellular carcinoma, LTC4S and NEUROD1 in lung adenocarcinoma (AUC = 0.998 and AUC = 0.994), FANCA and ARG2 in lung squamous cell carcinoma (AUC = 0.992 and AUC = 0.956).

The lowest AUC performance was achieved in prostate adenocarcinoma with AKR1B1 and CA12 (AUC = 0.659 and AUC = 0.651). The low performance in this dataset may be due to the low number of DEGs (1860,Table 3) obtained in DEA. Furthermore, the major issue in investigating this cancer was the low number of normal tissues for a comparison with tumour tissues in TCGA data (52 normal tissues).

PAFAH1B3 and AHCYL2 achieved the best AUC performance in rectum adenocarcinoma (AUC = 0.992), AKR1C4 and DNA2 in stomach adenocarcinoma (AUC = 0.983 and AUC = 0.98), GALE and SLC4A4 in thyroid carcinoma (AUC = 0.956 and AUC = 0.935); BRIP1 and GLI2 in uterine corpus endometrial carcinoma (AUC = 0.949).

PAFAH1B3 has been proposed as a driver cancer gene [46], while AHCYL2, which is highly expressed in the gastrointestinal tract [47], has been found to be highly downregulated in the gene expression profiling of colorectal tumour [48]. Despite being expressed specifically in the liver and stomach [47], AKR1C4 has been proposed as a possible target for cancer therapy in these tumours [49]. DNA2 nuclease has a role in the mechanism of double strand break repair and its mutation has been reported in gastric and colorectal carcinomas [50]. In malignant thyroid nodules, a different expression of GALE has been reported [51], while SLC4A4 has been included in a 15-gene profile proposed as diagnostic biomarkers of thyroid tumour [52].

## Discussion

The main limitation of current tools that analyze pathways, such as KEGG or Biocarta, is that they attribute the same role to each gene within a pathway in accomplishing the cellular function, without taking into account the effect of multiple gene interactions in performing that function.

Our approach considers, for what we believe is the first time, the integration of biological multiplex networks (such as physical interaction, genetic interaction, shared protein domains, co-localization, and functional reaction interactions) in pathways. Our method is based on a well-validated approach (the GANPA/LEGO method), based on the hypothesis that if one gene is functionally connected in the pathway with more genes than those expected (according to the functional networks), its role is functionally central in that pathway. Our approach is therefore an extension of the GANPA/LEGO method that defines key driver genes if they are highly connected in at least two interaction types simultaneously. We used this concept to reveal the gene drivers for multi pathways and networks, and developed a novel method for pan-cancer analysis. On the other hand, GANPA/LEGO [9, 10] was applied for expression-based gene set enrichment analysis. GANPA/LEGO attributes a weight for each gene in a pathway, based on the degree centrality index and its association with other genes inside and outside the pathway, according to the functional network. Weighted genes in pathways are then used to detect pathways with a significant expression change. To sum up, the aim of the two approaches is different, however the greatest difference of our approach is the multilayer analysis.

Since previous studies have shown that the absence of mutations in genes with a high degree centrality is vital for the organism survival [18], we found a list of 1322 network genes highly connected in at least two networks for each pathway, and we studied their behavior in 16 different types of cancer.

### Biological role of the key cancer driver DEGs

Our method was effective. Indeed, 67% (895/1322 genes) of the driver genes that we obtained were deregulated in at least one cancer type in TCGA data (Table 3). Our analysis identified several known cancer driver genes (Additional file 3), such as KRAS, PIK3CA, BRCA, BCL2, which regulate crucial pathways involved in apoptosis (i.e., BCL2, BAX, BCL2L1/11), hypoxia and energy metabolism (i.e., GNG4/7, ADCY5/7/8/9), angiogenesis (i.e., HLA-G/F, FGF5) and proliferation (i.e., BRIP1, BRCA1, TOPBP1, BLM) [13, 15]. Our method also revealed a lower probability of finding false positive DEGs than the DEG-based method alone, as demonstrated by the results shown in Fig. 4.

Of the 102 driver genes present in at least four networks (Fig. 3), we found that PSMA, PSMB, and PSMD were the cancer driver genes with a crucial role in the proteasome pathway (with also RPN1/2, PSMA3/5/6/7, PSMB2/3/4/8/9, PSMD2/3/4/7/11/12/14). Proteasome degradation is a crucial mechanism controlling abundance, protein aging and the activity of important protein regulators of cellular signal transduction including a variety of cellular proto-oncogenes [53]. Many preclinical studies have shown that proteasome inhibitors can induce apoptosis in cancer cell lines and murine models of cancer [54]. Unfortunately, however, chemoresistance to proteasome inhibitors can occur thus blocking its pharmacological activity [54]. Our approach could be useful to study driver genes of this pathway and to show how their deregulation in cancer can influence the entire pathway, thus suggesting a potential target of intervention to prevent and overcome chemoresistance.

In addition, among 102 genes, we found some genes belonging to POL and Period families (i.e. POLM, POLR3K, POLR2K) [55, 56], confirming the central role of DNA replication and the circadian rhythm in tumourigenesis [57], respectively.

Of the 895 genes, we found some genes of the mitochondrial respiration process (i.e., COX6A1, COX7A2). Mitochondrial energy metabolism is a well-known process that is altered in tumors [58] and among our 102 genes we have found several genes belonging to this pathway (i.e., GNGT1/2/11, GNG2). Energy metabolism has a high impact on other processes [59], such as apoptosis (BCL2, BCL2L1, BCL2L11), ROS production (i.e., COX5A) and cell cycle control (i.e., MCM protein family, RFC2/4/5).

Signal transduction genes (i.e., ADCY4/7/8/9,) are also in the list of our 102 genes, which also includes cancer stem cell signaling genes (i.e., WNT7A, GLI1/2/3, NOTCH2/3/4, ALDH1A3). Genome instability and DNA damage repair genes (i.e., XRCC protein family, PARP family member, FANC protein members), included in the list of our 102 genes, are among the genes that are altered in several cancers [60, 61].

**Fig. 7:**
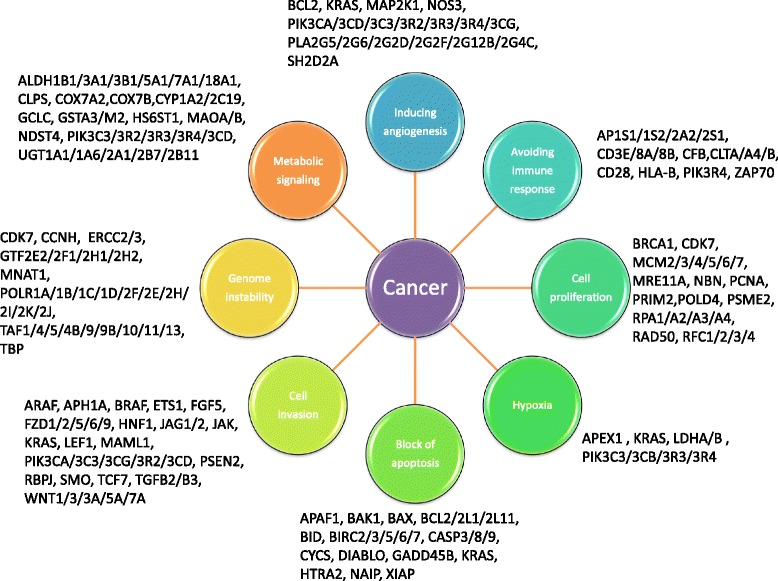
Cancer driver genes and cancer hallmarks obtained from KEGG and Reactome databases [5, 58, 59]

Looking for those genes with a role in the hallmarks of cancer [62], we generated Fig. 7, in which each circle represents one of the hallmark functions altered in cancer. The roles of genes in the hallmarks of cancer were obtained from KEGG and Reactome databases [5, 62, 63].

**Fig. 8:**
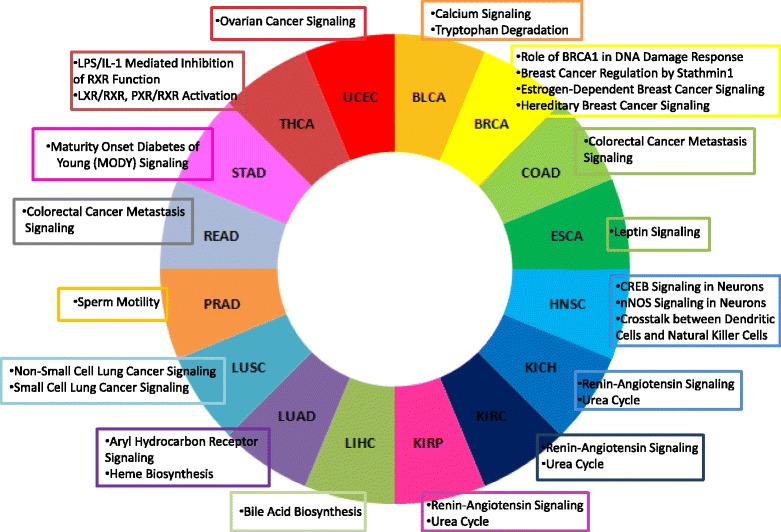
Cancer specific pathways enriched by cancer driver DEGs for each cancer

The list of cancer driver DEGs for each cancer also enabled us to highlight a series of pathways with a known cancer-specific role. Fig. 8 shows the cancer-specific pathways enriched by cancer driver DEGs for each cancer type considered in this study.

Our method identified 32 cancer driver DEGs which were found by none of the other 15 tools. These genes have important potential roles in metabolism (ALDH3B1, COX7A2, COX7B, GSTT1, IDO1, LSM7, LTC4S, PAFAH1B3, PNP, RPP25, TUBA1A), apoptosis (BIRC5, GADD45B), immune response (CFB, IDO1), DNA repair (DNA2), signal transduction (GAS1,GNG2, GNRH1, PPP1R1B) and proliferation (POLD4, PRIM2, PSME2, RPA3).

ALDH3B1 is an enzyme involved in the metabolism of endogenous and exogenous aldehydes and plays a critical role in maintaining cellular homeostasis [64]. ALDH proteins seem to have different, but not completely understood, roles in cancer. ALDH3B1 also acts against cellular oxidative stress by detoxifying aldehydes derived from ethanol metabolism and lipid peroxidation [64]. COX7A2 and COX7B, involved in energy metabolism, are components of the mitochondrial respiratory chain [65]. A correlation has been hypothesized between alterations in mitochondrial morphology and the reduced expression of COX7A2 in esophageal adenocarcinoma patients [65]. GSTT1 is involved in the metabolism of glutathione by catalyzing the detoxification of potential carcinogens [66]. Polymorphisms in this gene are associated with different types of cancer (e.g. oral, breast) [67, 68]. IDO1 is a catabolic enzyme involved in the pathways of tryptophan metabolism and plays a role in immune suppression [69] The increased expression of IDO1 in ovarian, endometrial and colorectal cancers has been associated with poor survival outcomes [69]. In addition, based on their immunosuppressive functions, IDO1 is becoming a potential target for drug discovery in cancer immunotherapy [70]. BIRC5 and GADD45B are involved in apoptosis, one of the most important hallmarks of cancer. They are known diagnostic, prognostic and therapeutic biomarkers in many tumours, such as gynecological, squamous cell carcinoma, and renal cell carcinoma [71–76]. POLD4, PRIM2, PSME2, and RPA3 are involved in cellular proliferation and genomic stability. Altered expression in these genes is associated with several tumours [77–81].

Overall, our approach detects both well-known cancer genes, as well as potential novel candidates.

### Comparison with other tools

Several approaches have been described to reveal cancer driver genes, but few methods have considered how the functional networks are affected by gene deregulation in cancer and have demonstrated how the integrative analysis of pathways and networks can be used effectively to identify key pathway patterns in cancer.

We compared our results with those obtained, in at least two methods, by 15 current different approaches and we found that almost 36.5% of our genes have been obtained by other methods. This thus suggests the reliability of our approach.

What makes our approach unique is its ability to identify the common/distinct biological processes involved in different cancer types. The above 15 algorithms mainly deal with the design of a static pathway by integrating genomic data, while our method enabled us to combine functional networks, based on multiple sources, with pathways and gene expression information.

**Fig. 9:**
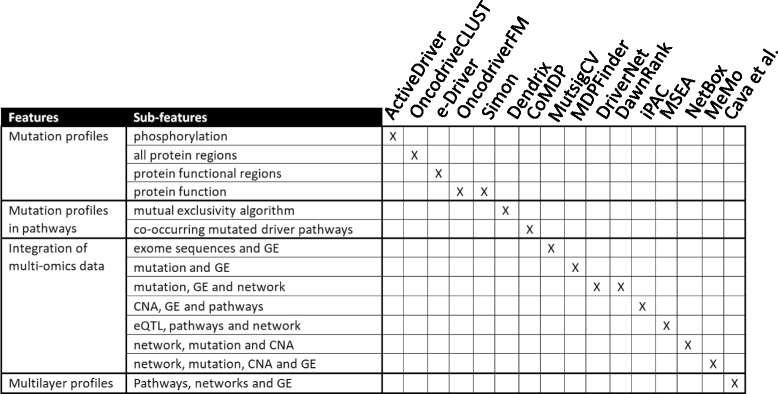
Comparison of different approaches used in our method and in 15 tools. GE: gene expression, CNA: copy number alteration, eQTL: expression quantitative trait loci

We grouped 15 current tools into three groups, based on: 1) mutation profiles, 2) mutation profiles in functional pathways, and 3) integration of multi-omics data (Fig. 9).

The first group includes ActiveDriver [27], OncodriveCLUST [41], e-Driver [38], OncodriveFM [32] and Simon [30]. With these tools cancer drivers were defined as 1) genes with unexpected mutation rates in phosphorylation-specific regions (ActiveDriver), 2) proteins with higher-than-expected mutation rates regardless of the protein regions (OncodriveCLUST), 3) proteins with somatic missense mutations in different protein functional regions, such as domains and intrinsically disordered regions (e-Driver), 4) genes analyzing the impact of mutations on the protein function (OncodriveFM and Simon). With respect to the above mentioned tools, our algorithm creates functional interactions between gene drivers and pathways. The integration of this information enables the genes to be selected that have a central role in biological processes and the generation of relationship among them.

The second group includes Dendrix [28] and CoMDP [35], two software tools that use an alternative approach to identify cancer driver genes: they examine mutations in the context of functional pathways. Compared to the tools in this group, our algorithm creates more functional interactions within pathways in order to obtain only a limited number of genes in the pathways that are highly connected.

The third group combines multi-omics data to overcome the mutational heterogeneity of cancer. This group includes MutsigCV [33], MDPFinder [29], DriverNet [37], DawnRank [36], iPAC [39], MSEA [40], NetBox [31] and MeMo [34]. MutsigCV was applied to exome sequences and gene expression levels, while MDPFinder is an integrative model of mutation and expression data used to identify biologically relevant gene sets. DriverNet associates the presence of a mutated gene with its influence on the gene expression levels of its known interacting genes. DawnRank detects personalized molecular drivers ranking potential driver genes on the basis of their impact on the overall differential expression of their downstream genes in the molecular interaction network. iPAC identifies driver genes with frequent copy-number alterations and corresponding changes in expression that are collectively enriched with respect to biological processes. MSEA integrates summary-level disease association data, functional genomics (such as expression quantitative trait loci, eQTLs, and ENCODE annotations), pathways, and gene networks to obtain disease-associated gene subnetworks and key regulatory genes. NetBox integrates the information of networks with sequence mutations and DNA copy number alterations. The underlying hypothesis of the NetBox tool is that gene networks are constituted by functional modules (sets of connected genes) critical for cancer hallmarks. The alterations of different combinations of genes can influence each module. NetBox identifies candidate driver mutations from perturbed modules. Similarly to NetBox, MeMo identifies candidate driver networks in cancers by focusing on modules that are recurrently altered and that exhibit patterns of mutually exclusive genetic alterations across multiple patients. Unlike Netbox, MeMo uses gene expression in addition to somatic mutations and copy numbers.

Compared to our approach, in the definition of cancer drivers, none of the existing methods are able to integrate different networks, pathways and gene expression in order to create a relationship among them. Our multi-layer profiles are able to extract for each driver, the information on the involved multi-pathways and multi-networks on regulatory pattern. To date the integration of multi-layer profiles has never been used to identify cancer driver genes.

Furthermore, the majority of the available methods use mutation data to detect cancer drivers, however this feature does not clarify the related molecular mechanisms. For example, mutations can exert very diverse effects, such as inducing a premature stop codon, reducing the dosage of mRNA transcripts or affecting the coding region of a gene, thus impacting on the protein function. However this does not necessarily mean that all abnormal genes are involved in the development of cancer. In fact, many aberrations have only mild or neutral effects, and the real drivers, i.e. those that promote the cancer phenotype, are only a minority.

## Conclusions

Gene signatures are often not reproducible in the sense that the inclusion or exclusion of a few patients can lead to different sets of selected genes which are difficult to interpret in a biological context. It is thus crucial to identify a limited number of genes that are central to the correct biological processes and which, if altered, can lead to pathological conditions.

To identify these genes, we have proposed an approach that integrates knowledge on the functional pathways and multiple gene-gene (protein-protein) interactions into gene selection algorithms. The challenge is to obtain more stable biomarker signatures, which are also more easily interpretable from a biological perspective.

The study of networks and pathways can also provide further hypotheses of the mechanisms of driver genes.

## Additional files

Additional file 1: Shows potential driver genes for each pathway and network. (XLSX 415 kb)

Additional file 2: Shows the results obtained by our method applied to the different networks identifying 1322 genes that are common in at least two networks. (XLSX 23 kb)

Additional file 3: Shows 895 cancer driver genes. (XLSX 18 kb)

Additional file 4: Shows cancer driver genes identified by our method (319/895) that were found in at least two of the different methods considered. (XLSX 10 kb)

## Abbreviations

AUC: Area Under Curve
DEA: differential expression analysis
FDR: False Discovery Rate
ROC: Receiver operating characteristic
